# Are Advanced Clinical Practitioners perfectly placed to re-report neuroimages to support clinical diagnosis of dementia?

**DOI:** 10.12968/ijap.2023.1.3.146

**Published:** 2023-10-02

**Authors:** Danielle Bradley, Joanna Harrison, Mark Goodall, Richard Dobrashian

**Affiliations:** Lancashire and South Cumbria NHS Foundation Trust; Synthesis Economic Evaluation and Decision Science (SEEDS) Group, University of Central Lancashire; Institute of Population Health, University of Liverpool; East Lancashire Hospitals NHS Trust

## Abstract

With the ageing population, the prevalence of dementia is increasing worldwide. There is an emphasis on early, timely diagnosis and treatment options for people with a dementia yet wait times from referral to diagnosis have increased. Neuroimaging performed by radiologists is utilised to support dementia diagnosis and some patients will already have a CT scan from a pre-existing condition such as stroke. The purpose of this commentary is to consider whether ACPs who specialise in dementia, are perfectly placed to re-report on pre-existing neuroimages to support the clinical diagnosis of dementia.

The purpose of this article is to consider whether Advanced Clinical Practitioners (ACPs) who specialise in dementia, are perfectly placed to re-report neuroimages of the brain to aid in the clinical diagnosis of dementia. An ACP in a Memory Assessment Service (MAS) has considered interpreting MRI and CT head scans, already performed for other clinical indications, with a specific focus on looking for signs of, and if present the potential subtype of dementia.

## Dementia prevalence and diagnostic assessment

Dementia is a progressive, clinical syndrome including impairments in memory, cognition, and neuro-behaviour which in turn interfere with functional activities of daily living. When two or more marked impairments are seen together, they may meet the diagnostic criteria for a dementia (WHO, 2023). The global prevalence of dementia in 2019 was estimated at 57.4 million cases, projected to increase to 152.8 million people in 2050 (GBD, 2022). In the UK alone, it was estimated there were approximately 900,000 older people living with dementia in 2019, projected to rise to approximately 1,600,000 in 2040 (Wittenberg et al. 2019), and 2.09 million in 2051 (Prince et al. 2014) With an ageing population, dementia is a public health concern and the anticipated increase in people being diagnosed will likely impact the health and social care system and cause a significant global socio-economic burden (El-Hayek et al. 2019). Dementia remains a health priority and the NHS Five Year Forward View continues to maintain a focus on diagnostic support for people with dementia and their carers (NHS England, 2017). Furthermore, the dementia wellbeing pathway (NHS England, 2022) identifies timely and accurate diagnosis as a standard within its transformation framework. The diagnostic process for a neurodegenerative disorder like dementia is multifactorial and determined by complex gene-environment interactions (Perneczky, 2019). For people diagnosed with dementia to receive the most appropriate treatment and support, it is imperative that a careful diagnostic assessment is undertaken (McCombe et al. 2022). Early diagnosis is valuable so that the patient and carer have access to treatments that can help manage symptoms and have time to plan for the future (Rasmussen et al. 2019).

NICE (2018) recommend that any patient with a suspected dementia undergo neuroimaging (structural imaging; looking at brain anatomy, structure and where the atrophy is) within the course of their diagnosis, mainly to exclude mimickers of dementia such as subdural hematomas, normal pressure hydrocephalus, and tumours. Further imaging should be considered if it would help diagnose a sub-type of dementia such as vascular, frontotemporal lobe or dementia in Lewy bodies (NICE 2018), and especially for Alzheimer’s disease where early treatment has been shown to slow down the progression of disease and improve quality of life (Dening et al. 2021). As with many illnesses, the diagnosis of dementia also benefits from a multidisciplinary approach. Within this, radiology plays an important role, supplementing the clinical impression with imaging and aiding diagnosis, especially in the clinically difficult early stages. Neuroimaging used in a multidisciplinary format also enables the clinician to refine their expertise, leading to a more informed diagnosis (Cronin et al. 2021).

## Current practice in referring to the Memory Assessment Services

To encourage early diagnosis and support for dementia, Memory Assessment Services (MAS) have been implemented in many high-income countries and consist of an integrated, multi-professional approach to diagnosis and management of dementia (Gomes et al. 2019). MAS however are not uniformed, meaning they work differently in many localities (Prince et al. 2016). In 2015, the UK government had an expectation that by 2020, an initial assessment for dementia would take place six weeks from the date of the GP referral (Department of Health [DOH], 2015). However, in the context of the pandemic, the average overall wait time from referral to diagnosis increased to 17.7 weeks (RCP, 2022). Interestingly, the Royal College of Psychiatrists (RCP 2022) also noted that out of 2750 CT or MRI scans requested for neuroimaging, 118 were not performed because a previous scan already existed. It is unclear if these existing scans were subsequently acquired for re-reporting.

## Innovative practice in ‘re-reporting’ of scans for dementia diagnosis

Re-reporting of neuroimages in patients with a suspected diagnosis of dementia consists of a Consultant Radiologist review of CT/MRI images which have already been performed, to rule out other organic pathologies, head injury or stroke. Re-reporting of these scans from a dementia perspective involves an in-depth focus on the memory specific areas of the brain such as the hippocampus, entorhinal cortex, septate nuclei and parietal lobes for atrophy and the contribution of vascular changes. Given that radiologists already experience greater demands on their time that take them away from direct clinical activities (RCR/SCR 2012) and with the volume of imaging needed in modern day medicine, any dementia reviews may be viewed as additional work. As an alternative to this process and to help relief pressure on radiologists, there is potential for extending the role of an ACP to review these scans following appropriate training and validation by a radiology professional. Advanced clinical practice is characterised by a high degree of autonomy and complex decision making which includes the analysis and synthesis of complex problems (HEE, 2017). It can therefore be argued that the skills of the ACP who specialise in dementia are well-suited to the task of re-reporting neuroimages.

The ACP of course is not a radiologist and has not undergone the same medical and radiological training. Re-reporting by an ACP would focus solely on supporting dementia diagnosis and not to identify coexistent organic pathology. Indeed, these scans will have been reported on previously by a Consultant Radiologist for that very purpose. However, there will be a mechanism to flag unexpected findings back to the supervising Consultant Radiologist for action if necessary. Double reading of screening is the standard of care in the National Health Service Breast Screening Program (Chen et al. 2023) and reporting by advanced practice radiographers is already incorporated into practice (Snaith et al. 2016). The Royal College of Radiologists (RCR) have formal standards for interpretation and state that reporting of imaging by non-radiologists should remain within their scope of practice, adhere to professional standards and work as part of a team with access to radiological opinion and advice (RCR 2018). ACPs would therefore require a robust training programme, mentoring, supervision and audit pathway to ensure the highest standards of the RCR are maintained. There are many PG Cert courses on image interpretation and a course could be provided by an accommodating university similar to the reporting courses provided for advanced practice radiographers.

## What is the evidence for interpretation of images by non-radiologists?

While there is no clear evidence base available for the ‘re-reporting’ of neuro-images, a number of systematic reviews have explored both ultra-sound (US) and computerised tomography (CT) interpretation by non-radiologists in a variety of healthcare indications. For instance, diagnostic accuracy of US was found to be similar between radiologist and non-radiologist for the detection of abdominal aortic aneurysms based on good quality evidence (Concannon et al. 2014). Likewise, the diagnostic accuracy of US for the characterisation of rotator cuff disorders (Roy et al. 2015) or the detection of lymph node metastasis (Issa et al. 2022) was similar whether performed by a trained radiologist or non-radiologist, based on studies where the overall summary of quality was unclear. In a scoping review, interpretation of US by non-radiologist personnel was also shown to have some positive impact on paediatric patient care such as reducing the number of CTs needed and reducing length of hospital stay (Van Wassenaer et al. 2021). Evidence for the interpretation of CT scans has reported more mixed results in two systematic reviews. Evans et al. (2017) found that emergency medical staff showed low concordance with the gold standard (radiologist review) and clinically significant misinterpretation when interpreting CT brain scans. Interpretation of CT spine scans was also found to have high discordance between non-radiologists and radiologists (Wani et al. 2023). However, both reviews were based on low to very low-quality evidence which limits confidence in their findings. Some primary studies have shown high concordance between radiologists and non-radiologists such as the reporting of abnormalities assessed with head CTs (Gallagher et al. 2011), interpretation of whole-body CT scans in trauma patients (Parag & Hardcastle 2020) and the interpretation of CT pulmonary angiograms for heart strain (Samaranayake et al. 2022). The evidence base for CT brain scan interpretation by non-radiologists is therefore not conclusive but recent single studies indicate potential.

## Can you train and support ACPs in ‘re-reporting’ neuroimages?

The RCR (2018) highlight that there are limitations in knowledge and training for allied health professionals when assessing images. Improving diagnostic accuracy is therefore important to consider in terms of both training and facilitating the re-reporting of scans by advanced clinical practitioners. There is currently no national curriculum or examination in place for non-doctors undertaking image reporting, and the type and length of training courses vary around the country (RCR, 2018). The Royal College of Nursing determine however, that a registered healthcare professional who is eligible to refer patients for clinical imaging must have developed their understanding of the relevant regulations through appropriate awareness training and experience, also engaging in continuing professional development and self-audit (RCN, 2021). Furthermore, the British Institute of Radiology strongly recommends that all non-medical referrers to radiology who review images and make decisions on patient treatment should receive appropriate training, including professional responsibilities and governance principles with mentorship by a consultant or GP (Hughes et al. 2022). This may be delivered in-house, via an educational institution, e-learning or a combination.

In addition to appropriate training, research has suggested that facilitators for interpreting CT images of the brain may be useful for improving diagnostic accuracy amongst non-radiologists. These include algorithmic approaches to neuroimaging in dementia, identifying imaging features with the greatest diagnostic value in dementia diagnosis (Harper et al. 2014), and the use of report templates in the diagnosis of brain injured patients (Evans et al. 2018). In other areas, diagnostic performance for detecting acute knee fractures was improved for non-radiologists when using colour coded reconstruction, enabling detecting of non-displaced fractures (Yang et al. 2020).

Looking to the future, using Artificial Intelligence (AI) innovations offers a potential way of supporting the radiology workload, like re-reporting of CT scans. Researchers have looked at the potential introduction of AI not in replacing the expert but in supporting their role, for example diagnostic imaging tasks (Gampala et al. 2020; Chen et al. 2021; Sorantin et al. 2022; Yang et al. 2022). However, although radiology is a forerunner in the use of AI innovations, there are issues still to be resolved before its use is routine. When asked, non-radiologists were more comfortable with an AI-generated radiologist confirmed diagnostic imaging method, rather than having just the AI-issued report alone (Lim et al. 2022). This adds to the other reported practical challenges: having condition specific validated algorithms; adopting early education, and ongoing training programmes; having plans for implementation and integration within clinical roles; developing ethical accountability strategies (Mazurowski et al. 2019; Chen et al. 2021; Schuur et al. 2021; Sorantin et al. 2022; Yang et al. 2022; Lim et al. 2022).

## ACPs could re-report neuroimages from a dementia perspective

The research evidence shows the potential for ACPs to action the re-reporting of neuroimages from a dementia perspective. ACPs could consider this as an enhancement of their skills, providing there is robust training, supervision and validation from the Consultant Radiologists. Moreover, this innovative way of working is the epitome of an ACP role, allowing for interpretation of relevant investigations, clinical reasoning and appropriate diagnosis. One might consider that re-reporting maybe viewed less favourably by ACPs if it thought to increase workload. Similarly, it could be perceived that radiologists may feel uncertainty about changes to the reporting process and providing training. Fellow ACPs in a local MAS however, have shown a keen interest in learning how to re-report neuroimages from a dementia perspective, with support and encouragement from the local radiologist for ACPs to acquire this skill. Furthermore, provision of training by a radiologist may impinge on time initially but with a view to releasing radiologist time in the long-term. Ultimately, these adaptations to practice could make a potential difference to the patient journey through reducing diagnostic delay and receiving access to timely treatment and support.

To our knowledge, there are no previous research studies of re-reporting by non-radiologists with a dementia specific focus. There is therefore a study underway to explore the interpretation of neuroimages by an ACP who specialises in dementia, with training, supervision and validation from a Consultant Radiologist. An ACP and Consultant Radiologist will review 300 neuroimages where the ACP should be at least 95% concordant with the consultant on dementia diagnosis. Research in this area will help to support the argument that ACPs who specialise in dementia are perfectly placed to re-report neuroimages, providing continuity of care and an opportunity to increase time and cost effectiveness within the Memory Assessment Services.

## Key Points

- The prevalence of dementia is increasing, and timely diagnosis remains important for patients and families to receive appropriate treatment and support.
- Neuroimaging is recommended for patients with suspected dementia to support diagnosis. Some patients will have CT images that were previously undertaken for conditions such as stroke.
- Re-reporting of CT images by Advanced Clinical Practitioners who specialise in dementia, with support from radiologists, may help with more timely dementia diagnosis within the Memory Assessment Services.
- Future research is needed to explore the interpretation of neuroimages by Advanced Clinical Practitioners, including concordance with radiologist review.
